# Osteopontin (OPN) as a CSF and blood biomarker for multiple sclerosis: A systematic review and meta-analysis

**DOI:** 10.1371/journal.pone.0190252

**Published:** 2018-01-18

**Authors:** Elmira Agah, Arshia Zardoui, Amene Saghazadeh, Mona Ahmadi, Abbas Tafakhori, Nima Rezaei

**Affiliations:** 1 Students’ Scientific Research Center, Tehran University of Medical Sciences, Tehran, Iran; 2 NeuroImmunology Research Association (NIRA), Universal Scientific Education and Research Network (USERN), Tehran, Iran; 3 Research Center for Immunodeficiencies (RCID), Children's Medical Center, Tehran University of Medical Sciences, Tehran, Iran; 4 Systematic Review and Meta-analysis Expert Group (SRMEG), Universal Scientific Education and Research Network (USERN), Boston, MA, United States of America; 5 Iranian Center of Neurological Research (ICNR), Tehran University of Medical Sciences, Tehran, Iran; 6 Network of Immunity in Infection, Malignancy and Autoimmunity (NIIMA), Universal Scientific Education and Research Network (USERN), Tehran, Iran; University of Münster, GERMANY

## Abstract

Identifying a reliable biomarker may accelerate diagnosis of multiple sclerosis (MS) and lead to early management of the disease. Accumulating evidence suggest that cerebrospinal fluid (CSF) and peripheral blood concentration of osteopontin (OPN) may have diagnostic and prognostic value in MS. We conducted a systematic review and meta-analysis of studies that measured peripheral blood and CSF levels of OPN in MS patients and controls to evaluate the diagnostic potential of this biomarker better. We searched PubMed, Web of Science and Scopus databases to find articles that measured OPN concentration in peripheral blood and CSF samples from MS patients up to October 19, 2016. Q statistic tests and the I2 index were applied for heterogeneity assessment. If the I2 index was less than 40%, the fixed-effects model was used for meta-analysis. Random-effects meta-analysis was chosen if the I2 value was greater than 40%. After removal of duplicates, 918 articles were identified, and 27 of them fulfilled the inclusion criteria. We included 22 eligible studies in the final meta-analysis. MS patients, in general, had considerably higher levels of OPN in their CSF and blood when compared to all types of controls (p<0.05). When the comparisons were made between different subtypes of MS patients and controls, the results pointed to significantly higher levels of OPN in CSF of MS subgroups (p<0.05). All subtypes of MS patients, except CIS patients, had increased blood levels of OPN compared to controls (p<0.05). In the second set of meta-analyses, we compared the peripheral blood and CSF concentrations of OPN between MS patient subtypes. CIS patients had significantly lower levels of OPN both in their peripheral blood and CSF compared to patients with progressive subtypes of MS (p<0.05). CSF concentration of OPN was significantly higher among RRMS patients compared to the CIS patients and SPMS patients (P<0.05). Finally, patients with active MS had significantly higher OPN levels in their CSF compared to patients with stable disease (P = 0.007). The result of this study confirms that increased levels of OPN exist in CSF and peripheral blood of MS patients and strengthens the evidence regarding the clinical utility of OPN as a promising and validated biomarker for MS.

## Introduction

Multiple sclerosis is a chronic, progressive, immune-mediated central nervous system (CNS) disorder characterized by inflammation, demyelination and axonal damage leading to neurodegeneration [1]. MS affects more than 2.5 million people worldwide [2], and is a leading cause of disability in young adults. According to their clinical course, MS patients are categorized into four major subtypes: (1) clinically isolated syndrome (CIS); an initial presentation of MS, (2) relapsing-remitting MS (RRMS); the most common type of MS, (3) primary progressive MS (PPMS); clinically progressive disease without any recovery, and (4) secondary progressive MS (SPMS); which usually develops after years of relapsing-remitting disease [3].

The heavy burden of disease necessitates early diagnosis and management of MS [4]. However, correct diagnosis of MS may be challenging, especially during the initial stages in which individuals may present with more non-specific complaints and imaging signs [5]. According to the McDonald diagnostic criteria, for true differentiation of MS from other alternative diagnoses, patient brain lesions need to fulfill the dissemination in time and space conditions [6]. Although brain magnetic resonance imaging (MRI) has a critical role in the diagnosis of MS [7], overlap between MRI findings of MS and other neurological disorders makes a definite diagnosis of MS difficult [8]. Evaluation of the cerebrospinal fluid (CSF) and evoked potential (EP) studies might be helpful in these cases [9, 10]. EPs are less sensitive than MRI but may be beneficial for diagnosing MS in subclinical cases [10]. Analysis of CSF for immunoglobulin G (IgG) index and oligoclonal bands (OCBs) can provide diagnostic aid in suspected cases of MS [6, 9]. However, lumbar puncture (LP), which is used to collect CSF, is an invasive procedure that might limit the applicability of these tests. Since blood collection is a less invasive procedure, finding a reliable blood-borne biomarker for MS is urgently needed [5, 11, 12].

Biomarkers are objective indicators of underlying pathology [13]; and they could be applied for diagnostic, prognostic and therapeutic aims in the clinical setting [11, 12, 14]. A variety of molecular biomarkers have been proposed for MS; however, a minority of them have been employed in clinical practice [12]. Based on evidence strength, proposed biomarkers for MS are categorized into exploratory or potential biomarkers, validated biomarkers, and clinically useful biomarkers [11]. The category of validated biomarkers has good potential to become clinically useful. To achieve this goal, critical evaluation of existing evidence is needed. Currently, this category of biomarkers is mostly composed of inflammatory biomarkers such as interleukin 17 (IL-17), the tumor necrosis factor (TNF) superfamily, and osteopontin (OPN) [11, 12, 15].

OPN is an extracellular matrix protein involved in a variety of physiologic functions and pathological states such as bone remodeling, wound healing, cancer biology, vascular disorders, and inflammatory diseases [15, 16]. OPN is widely expressed in immune cells, including T cells, dendritic cells, macrophages, and natural killer cells and contributes to inflammation via increasing production of IL-12, IL-17 and interferon gamma (IFN-γ) and inhibiting expression of IL-10 [17]. Therefore, its potential role in pathology of MS as an autoimmune disorder has frequently been investigated [18–20]. The OPN gene expression was found to be increased in MS brain lesions [21]. Different studies have found that the variant of OPN gene has a significant impact on risk of developing MS, the disease course, and serum OPN levels [22, 23]. Also, some other studies in both humans and animals have linked the OPN levels to disease progression and recurrent relapses [19, 24]. Therefore, OPN may be a good disease biomarker for MS. Thus far, altered levels of OPN in blood and CSF of MS patients have been suggested by many studies and majority report increased concentrations of OPN in patients with MS [20, 22, 25–49]. However, as disease controls, changes in OPN level in other inflammatory and non-inflammatory conditions have also been observed. So, there is a need to appraise and systematically review the existing literature.

To the best of our knowledge, this study is the first systematic review and meta-analysis of studies that have measured peripheral blood and CSF levels of OPN in MS patients and controls. The aim of the present meta-analysis is to evaluate the potential of OPN as a diagnostic biomarker for MS.

## Materials and methods

### Search strategy and study selection

This paper was written according to the preferred reporting items for systematic reviews and meta-analyses (PRISMA) statement (S1 File) [50]. The protocol of this systematic review and meta-analysis was registered in the PROSPERO website (https://www.crd.york.ac.uk/PROSPERO/) with registration number CRD42016043050.

We searched PubMed, Web of Science and Scopus databases to find related articles up to October 19, 2016. Combination of the following terms was used to identify eligible studies: (“Osteopontin” OR “OPN” OR “Bone sialoprotein I” OR “BSP-1” OR “BSP 1” OR “BSP1” OR “BSPI” OR “BNSP” OR “Early T-lymphocyte activation” OR “ETA-1” OR “ETA 1” OR “ETA1” OR “ETAI” OR “Secreted phosphoprotein 1” OR “SPP-1” OR “SPP 1” OR “SPP1” OR “SPPI” OR “Rickettsia resistance” OR “Ric” AND (Multiple sclerosis OR MS OR Disseminated sclerosis OR Encephalomyelitis disseminata). A detailed search strategy is provided in S1 Appendix. Reference lists of the relevant articles were searched manually to identify additional related studies. All literature searches were performed by two authors independently (EA and AS) and no language or publication date restriction was applied.

Original articles were considered eligible to be included if: (1) they were observational studies or reported the baseline phase of randomized clinical trials (RCTs) which measured OPN in CSF or blood samples of human subjects diagnosed with MS; (2) sufficient data (at least number of patients and study controls as well as the OPN numerical measurement results) were provided. Studies were not considered if no comparison group existed or if any history of concomitant disease or any drug use, which may affect the concentration of OPN, was noted.

### Quality assessment and data extraction

Two of our authors (EA and AZ) extracted the following data from each study individually: First author’s name, year of publication, country, sample type (CSF or peripheral blood), number of participants, age, sex ratio, duration of disease (year), MS subtype, type of control group, MS phase at time of sampling (active/stable or relapse/remission), treatment status at sampling (treated or untreated) and OPN levels. Third author’s (AS or AT) opinion was sought for any inconsistency found among the data extracted.

For quality appraisal of included studies, Newcastle–Ottawa Scale (NOS) for nonrandomized studies was used.[51] This tool evaluates the risk of bias by assessing the quality of sample selection, comparability of cases and controls and the outcome ascertainment method. Then, studies are judged as high-quality (with a score range of 7–9), medium-quality (scores of 4–6) or low-quality (scores less than 4) based on the scores obtained (possible range: 0–9).

In all meta-analyses, MS patients (CIS, RRMS, SPMS, PPMS or unspecified) were included as the disease group. To better compare the cases and controls, control subjects were further categorized as; healthy controls (HCs), non-inflammatory neurological disorder (NIND) controls, and inflammatory neurological disorder (IND) controls.

### Statistical analysis

All meta-analyses were performed by using the STATA version 14.0. Mean and standard deviation (SD) of OPN concentrations and the number of participants were also entered for each group. Regarding the studies which reported the median and range/inter-quartile range of OPN, mean SD was estimated if the sample size was reported; otherwise, it was excluded. Q statistic tests and the I^2^ index were applied for heterogeneity assessment. The I^2^ index was interpreted according to the Cochrane handbook [52]. If the I^2^ index was less than 40%, the heterogeneity was considered not important; therefore, fixed-effects model was used for meta-analysis. Random-effects meta-analysis was chosen if the I^2^ value was greater than 40%. Standardized mean difference (SMD) and 95% confidence interval (CI) were used for effect measurement. Publication bias was evaluated by funnel plot and Egger test if more than 4 studies were included in the meta-analysis [53]. In this study, p values ≤ 0.05 are considered significant.

## Results

**Fig 1:**
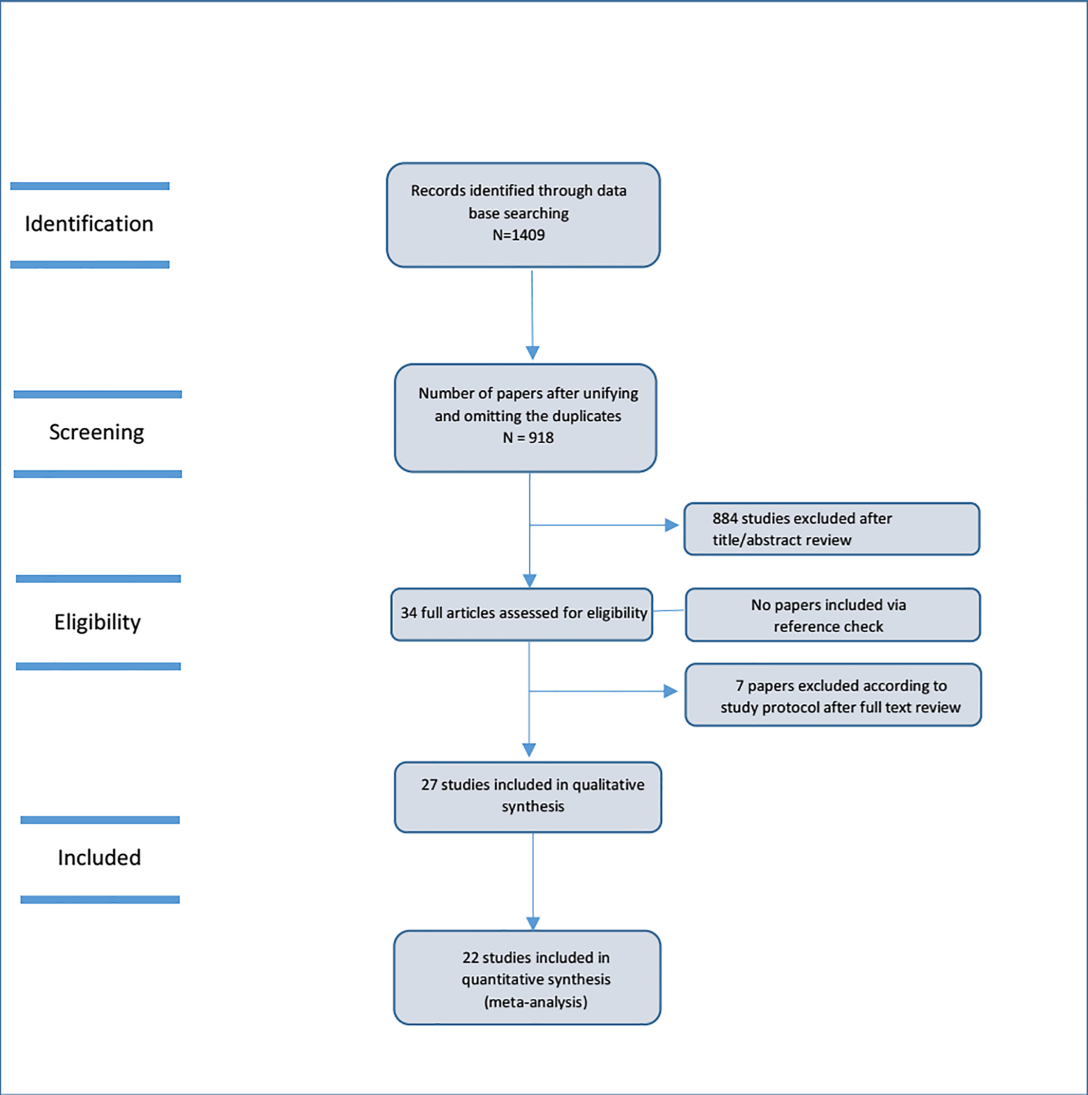
Flow diagram of studies.

Our search strategy identified 1,409 articles initially, and, after removal of duplicates, 918 articles remained. The remaining articles were assessed for eligibility, and 27 of them fulfilled the inclusion criteria. We later searched 98 pages of Google Scholar to find additional articles, but no paper was added. Finally, 22 studies were included in the meta-analysis (five studies were excluded due to not reporting sufficient data) (Fig 1). Characteristics of the included studies are shown in the S1 Table. The results of quality assessment showed a medium quality for eight articles and high quality for rest of the studies included in the meta-analysis (Table 1).

**Table 1:** Quality assessment of the included studies.

Study	Selection				Comparability		Exposure			Score
**Vogt 2003**		*		*	*	*	*	*	*	7
**Chiocchetti 2005**	*	*	*	*	*		*	*	*	8
**Chowdhury 2008**	*			*	*		*	*	*	6
**Braitch 2008**				*	*		*	*	*	5
**Khademi 2009**	*	*	*	*			*	*	*	7
**Altıntaş 2009**	*			*	*	*	*	*	*	7
**Vogt 2010**	*	*		*	*	*	*	*	*	8
**Bornsen 2011**				*	*	*	*	*	*	6
**Assadi 2011**	*	*	*	*	*	*	*	*	*	9
**Wen 2012**	*			*	*	*	*	*	*	7
**Romme Christensen 2013**	*	*		*	*		*	*	*	7
**Szalardy 2013**	*			*	*	*	*	*	*	7
**Shimizu 2013**				*	*		*	*	*	5
**Iaffaldano 2013**				*	*	*	*	*	*	6
**Edwards 2013**		*		*	*	*	*	*	*	7
**Khademi 2013**	*	*	*	*	*	*	*	*	*	9
**Kivisäkk 2104**	*	*		*	*	*	*	*	*	8
**Ma 2014**				*	*		*	*	*	5
**Stilund 2015**	*	*		*	*	*	*	*	*	8
**Kariya 2015**				*	*	*	*	*	*	6
**Strehlow 2016**				*	*	*	*	*	*	6
**Ferret-Sena 2016**	*	*		*	*	*	*	*	*	8

**Fig 2:**
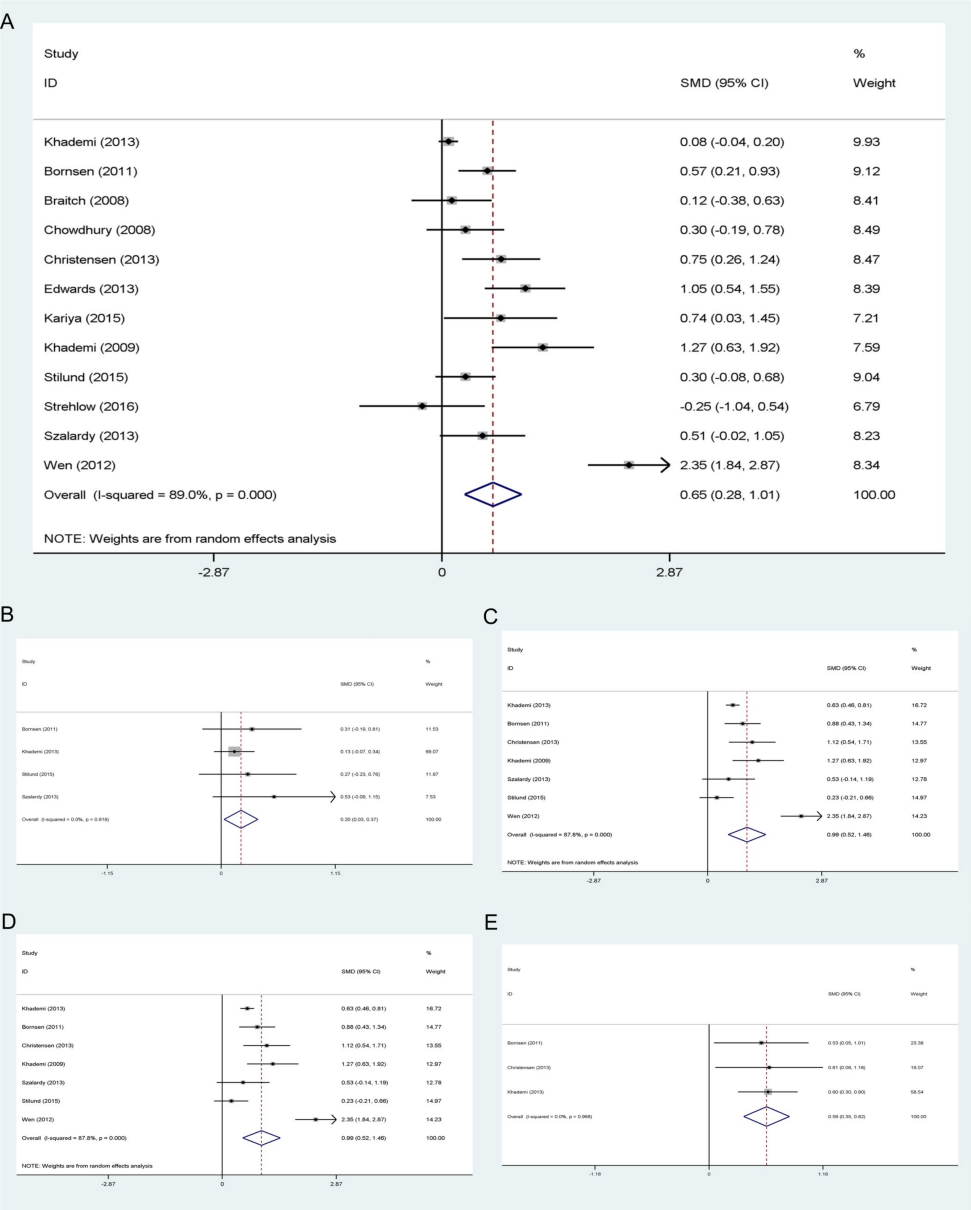
CSF concentration of OPN in MS patients compared to controls. (A) MS patients compared to all controls. (B) CIS patients compared to HCs and NIND patients. (C) RRMS patients compared to HCs and NIND patients. (D) PPMS patients compared to HCs and NIND patients. (E) SPMS patients compared to NIND patients.

**Fig 3:**
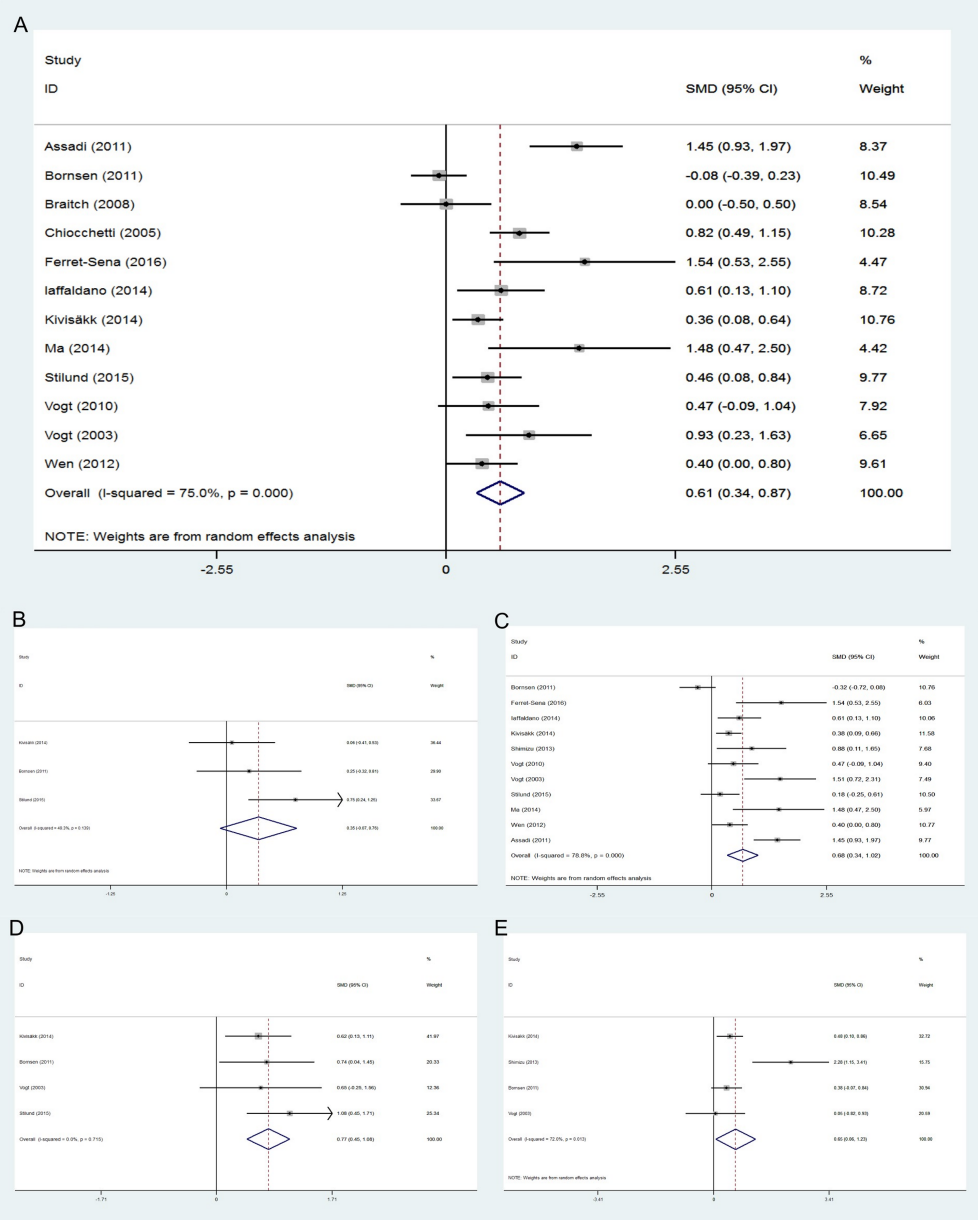
Meta-analysis of peripheral blood OPN levels: MS patients compared to controls. (A) MS patients versus all controls. (B) CIS patients versus HCs. (C) RRMS patients versus HCs and NIND patients. (D) PPMS patients versus HCs and NIND patients. (E) SPMS patients versus HCs and NIND patients.

First, we conducted a set of meta-analyses to find the difference between MS patients and controls regarding both peripheral blood (plasma or serum) and CSF concentration of OPN (a full summary of results is shown in Tables 2 and 3). MS patients, in general, had a considerably higher level of OPN in their CSF compared to all types of controls (p<0.01, Fig 2 (A)). When the comparisons were made between different subtypes of MS patients and controls, the results pointed to significantly higher levels of OPN in CSF of MS subgroups (Fig 2). Blood concentrations of OPN were also significantly higher in MS patients (p<0.05), however, Q statistic results revealed a considerable heterogeneity among the studies which measured the OPN level in peripheral blood (more than 90%). We realized that one of the included studies [25], is a major source of heterogeneity, so we omitted it from all meta-analyses with I^2^ index> 75% to get more reliable results. All subtypes of MS patients except CIS patients had increased blood levels of OPN compared to the HCs and/or NIND patients (p<0.05, Fig 3).

**Table 2:** Summary of meta-analyses undertaken in studies which measured CSF levels of OPN.

Meta-analysis			No of comparisons	No of group 1/ group 2	Heterogeneity chi2	Inconsistency	Effect measure (95% CI)	Overall effect (p value)	Egger’s test	References
Specimen	Group I	Group II								
CSF	MS	IND	4	715/248	*X*^2^ = 8.22 *P* = 0.042	*I*^2^ = 63.5%	SMD = -0.476 (-1.007–0.055)	Z = 1.76 *P* = 0.079	N/A	[28, 32, 37, 45]
CSF	MS	NIND	9	1032/414	*X*^2^ = 79.47 *P* < 0.001	*I*^2^ = 89.9%	SMD = 1.137 (0.661–1.613)	Z = 4.68 *P* < 0.001	T = 2.30 *P* = 0.055	[27, 28, 32, 36, 37, 41, 45, 46, 49]
CSF	MS	NIND and HC	10	1118/466	*X*^2^ = 78.80 *P* < 0.001	*I*^2^ = 89.5%	SMD = 0.921 (0.499–1.343)	Z = 4.28 *P* < 0.001	T = 1.52 *P* = 0.166	[27, 28, 32, 36, 37, 41, 44–46, 49]
CSF	MS	IND/NIND	11	1081/712	*X*^2^ = 98.67 *P* < 0.001	*I*^2^ = 89.9%	SMD = 0.699 (0.296–1.103)	Z = 3.40 *P* = 0.001	T = 2.43 ***P* = 0.038**	[27–29, 32, 35–37, 41, 45, 46, 49]
CSF	MS	IND/NIND and HC	12	1167/764	*X*^2^ = 100.02 *P* < 0.001	*I*^2^ = 89.0%	SMD = 0.645 (0.285–1.006)	Z = 3.51 *P* < 0.001	T = 2.37 ***P* = 0.039**	[27–29, 32, 35–37, 41, 44–46, 49]
CSF	MS (untreated*)	NIND	5	293/132	*X*^2^ = 4.05 *P* = 0.400	*I*^2^ = 1.2%	SMD = 0.705 (0.485–0.924)	Z = 6.29 *P* < 0.001	T = 1.68 *P* = 0.191	[27, 28, 36, 41, 46]
CSF	MS (untreated*)	NIND and HC	6	379/171	*X*^2^ = 7.35 *P* = 0.196	*I*^2^ = 32.0%	SMD = 0.603 (0.413–0.793)	Z = 6.22 *P* < 0.001	T = 2.35 *P* = 0.079	[27, 28, 36, 41, 44, 46]
CSF	MS (untreated*)	IND/NIND	5	293/143	*X*^2^ = 8.05 *P* = 0.090	*I*^2^ = 50.3%	SMD = 0.612 (0.297–0.926)	Z = 3.82 *P* < 0.001	T = 0.72 *P* = 0.522	[27, 28, 36, 41, 46]
CSF	MS (untreated*)	IND/NIND and HC	6	379/182	*X*^2^ = 9.78 *P* = 0.082	*I*^2^ = 48.9%	SMD = 0.545 (0.276–0.814)	Z = 3.97 *P* < 0.001	T = 1.15 *P* = 0.314	[27, 28, 36, 41, 44, 46]
CSF	CIS	NIND and HC	4	243/305	*X*^2^ = 1.79 *P* = 0.618	*I*^2^ = 0.0%	SMD = 0.199 (0.029–0.369)	Z = 2.30 *P* = 0.022	N/A	[27, 37, 44, 46]
CSF	RRMS	NIND	6	551/359	*X*^2^ = 42.20 *P* < 0.001	*I*^2^ = 88.2%	SMD = 1.124 (0.590–1.658)	Z = 4.13 *P* < 0.001	T = 1.42 *P* = 0.229	[27, 36, 37, 41, 46, 49]
CSF	RRMS	NIND and HC	7	595/398	*X*^2^ = 49.26 *P* < 0.001	*I*^2^ = 87.8%	SMD = 0.989 (0.516–1.463)	Z = 4.09 *P* < 0.001	T = 1.08 *P* = 0.328	[27, 36, 37, 41, 44, 46, 49]
CSF	RRMS (untreated*)	NIND and HC	5	155/147	*X*^2^ = 10.41 *P* = 0.034	*I*^2^ = 61.6%	SMD = 0.780 (0.387–1.173)	Z = 3.89 *P* < 0.001	T = 1.13 *P* = 0.340	[27, 36, 41, 44, 46]
CSF	PPMS	NIND	4	68/286	*X*^2^ = 14.89 *P* = 0.002	*I*^2^ = 79.9%	SMD = 1.183 (0.448–1.919)	Z = 3.15 *P* = 0.002	N/A	[27, 37, 41, 46]
CSF	PPMS	NIND and HC	5	83/325	*X*^2^ = 15.43 *P* = 0.004	*I*^2^ = 74.1%	SMD = 1.056 (0.494–1.619)	Z = 4.59 *P* < 0.001	T = 3.05 *P* = 0.056	[27, 37, 41, 44, 46]
CSF	SPMS	NIND	3	122/267	*X*^2^ = 0.07 *P* = 0.968	*I*^2^ = 0.0%	SMD = 0.586 (0.353–0.819)	Z = 4.93 *P* < 0.001	N/A	[27, 37, 41]
CSF	Progressive MS	NIND	4	190/286	*X*^2^ = 2.44 *P* = 0.487	*I*^2^ = 0.0%	SMD = 0.628 (0.426–0.826)	Z = 6.13 *P* < 0.001	N/A	[27, 37, 41, 46]
CSF	Progressive MS	NIND and HC	5	205/325	*X*^2^ = 2.44 *P* = 0.655	*I*^2^ = 0.0%	SMD = 0.882 (0.437–0.818)	Z = 6.47 *P* < 0.001	N/A	[27, 37, 41, 44, 46]
CSF	CIS	RRMS	4	243/488	*X*^2^ = 5.42 *P* = 0.143	*I*^2^ = 44.7%	SMD = -0.360 (-0.639 –-0.081)	Z = 2.53 *P* = 0.011	N/A	[27, 37, 44, 46]
CSF	CIS	PPMS	4	243/62	*X*^2^ = 1.56 *P* = 0.669	*I*^2^ = 0.0%	SMD = -0.435 (-0.723 –-0.146)	Z = 2.95 *P* = 0.003	N/A	[27, 37, 44, 46]
CSF	CIS	Progressive MS	4	243/144	*X*^2^ = 0.58 *P* = 0.900	*I*^2^ = 0.0%	SMD = -0.421 (-0.634 - -0.208)	Z = 3.87 *P* < 0.001	N/A	[27, 37, 44, 46]
CSF	RRMS	PPMS	5	524/83	*X*^2^ = 6.41 *P* = 0.171	*I*^2^ = 37.6%	SMD = 0.088 (-0.158–0.334)	Z = 0.70 *P* = 0.485	T = -1.42 *P* = 0.252	[27, 37, 41, 44, 46]
CSF	RRMS	SPMS	3	463/96	*X*^2^ = 2.39 *P* = 0.303	*I*^2^ = 16.2%	SMD = 0.342 (0.089–0.595)	Z = 2.65 *P* = 0.008	N/A	[27, 37, 41]
CSF	RRMS	Progressive MS	5	524/205	*X*^2^ = 8.88 *P* = 0.064	*I*^2^ = 55.0%	SMD = 0.142 (-0.158–0.443)	Z = 0.93 *P* = 0.353	T = -0.77 *P* = 0.497	[27, 37, 41, 44, 46]
CSF	SPMS	PPMS	3	96/58	*X*^2^ = 4.95 *P* = 0.084	*I*^2^ = 59.6%	SMD = -0.227 (-0.771–0.318)	Z = 0.82 *P* = 0.414	N/A	[27, 37, 41]
CSF	Active MS	Stable MS	3	103/333	*X*^2^ = 3.14 *P* = 0.208	*I*^2^ = 36.3%	SMD = 0.323 (0.090–0.556)	Z = 2.71 *P* = 0.007	N/A	[29, 37, 46]

*patients who were drug naïve or samples were collected after the washout period.

**Table 3:** Summary of meta-analyses undertaken in studies which measured peripheral blood levels of OPN.

Meta-analysis			No of comparisons	No of group 1/ group 2	Heterogeneity chi2	Inconsistency	Effect measure (95% CI)	Overall effect (p value)	Egger’s test	References
Specimen	Group I	Group II								
Plasma	MS	HC	6	760/138	*X*^2^ = 7.55 *P* = 0.183	*I*^2^ = 33.7%	SMD = 0.480 (0.291–0.669)	Z = 4.97 *P* <0.001	T = 3.30 ***P* = 0.030**	[20, 27, 33, 34, 38, 48]
Serum	MS	HC	4	202/168	*X*^2^ = 10.67 *P* = 0.014	*I*^2^ = 71.9%	SMD = 0.960 (0.500–1.420)	Z = 4.09 *P* <0.001	N/A	[22, 26, 39, 44]
Serum/Plasma	MS	HC	10	962/306	*X*^2^ = 24.79 *P* = 0.003	*I*^2^ = 63.7%	SMD = 0.723 (0.464–0.981)	Z = 5.47 *P* <0.001	T = 2.16 *P* = 0.063	[20, 22, 26, 27, 33, 34, 38, 39, 44, 48]
Plasma	MS (untreated*)	HC	4	256/109	*X*^2^ = 5.13 *P* = 0.163	*I*^2^ = 41.5%	SMD = 0.591 (0.262–0.920)	Z = 3.52 *P* <0.001	N/A	[27, 33, 34, 38]
Serum/Plasma	MS (untreated*)	HC	6	352/158	*X*^2^ = 8.89 *P* = 0.113	*I*^2^ = 43.8%	SMD = 0.609 (0.326–0.892)	Z = 4.22 *P* <0.001	T = 3.40 ***P* = 0.027**	[27, 33, 34, 38, 39, 44]
Serum/Plasma	MS	NIND	3	175/116	*X*^2^ = 4.75 *P* = 0.093	*I*^2^ = 57.9%	SMD = 0.072 (-0.308–0.452)	Z = 0.37 *P* = 0.710	N/A	[27, 28, 49]
Plasma	MS	NIND and HC	7	787/206	*X*^2^ = 14.40 *P* = 0.025	*I*^2^ = 58.3%	SMD = 0.432 (0.148–0.716)	Z = 2.98 *P* = 0.003	T = 2.13 *P* = 0.086	[20, 27, 28, 33, 34, 38, 48]
Serum	MS	NIND and HC	5	253/216	*X*^2^ = 15.11 *P* = 0.004	*I*^2^ = 73.5%	SMD = 0.821 (0.419–1.223)	Z = 4.00 *P* <0.001	T = 1.18 *P* = 0.323	[22, 26, 39, 44, 49]
Serum/Plasma	MS	NIND and HC	12	1040/422	*X*^2^ = 43.3 P <0.001	*I*^2^ = 74.6%	SMD = 0.609 (0.342–0.876)	Z = 4.47 P <0.001	T = 2.1 *P* = 0.062	[20, 22, 26–28, 33, 34, 38, 39, 44, 48, 49]
Serum/Plasma	MS (Stable^†^)	NIND and HC	5	131/123	*X*^2^ = 11.59 *P* = 0.021	*I*^2^ = 65.5%	SMD = 0.679 (0.214–1.143)	Z = 2.86 *P* = 0.004	T = 1.92 *P* = 0.150	[28, 33, 34, 43, 49]
Serum/Plasma	MS (untreated*)	IND/NIND and HC	7	330/237	*X*^2^ = 22.75 *P* = 0.001	*I*^2^ = 73.6%	SMD = 0.520 (0.151–0.889)	Z = 2.76 *P* = 0.006	T = 2.46 *P* = 0.057	[27, 28, 33, 34, 38, 39, 44]
Serum/Plasma	MS	IND/NIND	3	175/127	*X*^2^ = 4.78 *P* = 0.092	*I*^2^ = 58.1%	SMD = 0.066 (-0.304–0.437)	Z = 0.35 *P* = 0.725	N/A	[27, 28, 49]
Serum/Plasma	MS	IND/NIND and HC	12	1040/433	*X*^2^ = 44.08 *P <0*.*001*	*I*^2^ = 75.0%	SMD = 0.606 (0.338–0.873)	Z = 4.44 P <0.001	T = 2.16 *P* = 0.056	[20, 22, 26–28, 33, 34, 38, 39, 44, 48, 49]
Serum/Plasma	MS (stable^†^)	IND/NIND and HC	5	180/134	*X*^2^ = 12.22 *P* = 0.016	*I*^2^ = 67.3%	SMD = 0.639 (0.193–1.084)	Z = 2.81 *P* = 0.005	T = 2.05 *P* = 0.133	[28, 33, 34, 43, 49]
Serum/Plasma	MS (untreated*)	IND/NIND and HC	7	330/237	*X*^2^ = 22.75 *P* = 0.001	*I*^2^ = 73.6%	SMD = 0.520 (0.151–0.889)	Z = 2.76 *P* = 0.006	T = 2.46 *P* = 0.057	[27, 28, 33, 34, 38, 39, 44]
Serum/Plasma	CIS	HC	3	77/127	*X*^2^ = 3.94 *P* = 0.139	*I*^2^ = 49.3%	SMD = 0.347 (-0.069–0.763)	Z = 1.64 *P* = 0.102	N/A	[27, 38, 44]
Serum/Plasma	CIS	NIND and HC	3	77/161	*X*^2^ = 5.83 *P* = 0.054	*I*^2^ = 65.7%	SMD = 0.244 (-0.229–0.717)	Z = 1.01 *P* = 0.313	N/A	[27, 38, 44]
Plasma	RRMS	HC	7	588/158	*X*^2^ = 18.59 *P* = 0.005	*I*^2^ = 67.7%	SMD = 0.632 (0.257–1.008)	Z = 3.30 *P* = 0. 001	T = 1.84 *P* = 0.125	[20, 27, 33, 34, 38, 43, 48]
Serum	RRMS	HC	3	89/87	*X*^2^ = 15.60 *P <0*.*001*	*I*^2^ = 87.2%	SMD = 0.993 (0.022–1.965)	Z = 2.01 *P* = 0.045	N/A	[26, 39, 44]
Serum/Plasma	RRMS	HC	10	677/245	*X*^2^ = 36.59 *P <0*.*001*	*I*^2^ = 75.4%	SMD = 0.741 (0.384–1.098)	Z = 4.07 P <0.001	T = 2.15 *P* = 0.064	[20, 26, 27, 33, 34, 38, 39, 43, 44, 48]
Plasma	RRMS (remission)	HC	7	588/158	*X*^2^ = 18.59 *P* = 0.005	*I*^2^ = 67.7%	SMD = 0.632 (0.257–1.008)	Z = 3.30 P = 0.001	T = 1.84 *P* = 0.125	[20, 27, 33, 34, 38, 43, 48]
Plasma	RRMS (untreated*)	HC	4	197/109	*X*^2^ = 10.86 *P* = 0.013	*I*^2^ = 72.4%	SMD = 0.535 (0.029–1.041)	Z = 2.07 *P* = 0.038	N/A	[27, 33, 34, 38]
Serum/Plasma	RRMS (untreated*)	HC	6	251/158	*X*^2^ = 16.35 *P* = 0.006	*I*^2^ = 69.4%	SMD = 0.555 (0.147–0.964)	Z = 2.66 *P* = 0.008	T = 1.40 *P* = 0.234	[27, 33, 34, 38, 39, 44]
Plasma	RRMS	NIND and HC	7	588/202	*X*^2^ = 27.22 *P <0*.*001*	*I*^2^ = 78.0%	SMD = 0.618 (0.186–1.050)	Z = 2.81 *P* = 0. 005	T = 2.01 *P* = 0.101	[20, 27, 33, 34, 38, 43, 48]
Serum	RRMS	NIND and HC	4	140/135	*X*^2^ = 17.71 *P* = 0.001	*I*^2^ = 83.1%	SMD = 0.808 (0.164–1.451)	Z = 2.46 *P* = 0.014	N/A	[26, 39, 44, 49]
Serum/Plasma	RRMS	NIND and HC	11	728/337	*X*^2^ = 47.10 *P <0*.*001*	*I*^2^ = 78.8%	SMD = 0.682 (0.340–1.024)	Z = 3.90 P <0.001	T = 2.56 ***P* = 0.031**	[20, 26, 27, 33, 34, 38, 39, 43, 44, 48, 49]
Serum/Plasma	RRMS (remission)	NIND and HC	3	87/79	*X*^2^ = 4.49 *P* = 0.106	*I*^2^ = 55.5%	SMD = 0.736 (0.198–1.274)	Z = 2.68 *P* = 0.007	N/A	[33, 34, 49]
Serum/Plasma	RRMS (untreated*)	NIND and HC	6	251/202	*X*^2^ = 24.20 *P <0*.*001*	*I*^2^ = 79.3%	SMD = 0.540 (0.069–1.012)	Z = 2.25 *P* = 0.025	T = 1.73 *P* = 0.159	[27, 33, 34, 38, 39, 44]
Plasma	SPMS	HC	4	96/108	*X*^2^ = 12.40 *P* = 0.006	*I*^2^ = 75.8%	SMD = 0.879 (0.186–1.572)	Z = 2.49 *P* = 0.013	N/A	[20, 27, 38, 43]
Plasma	SPMS	NIND and HC	4	96/152	*X*^2^ = 10.71 *P* = 0.013	*I*^2^ = 72.0%	SMD = 0.646 (0.058–1.234)	Z = 2.15 *P* = 0.031	N/A	[20, 27, 38, 43]
Plasma	PPMS	HC	3	43/88	*X*^2^ = 2.90 *P* = 0.234	*I*^2^ = 31.1%	SMD = 0.798 (0.412–1.183)	Z = 4.06 P <0.001	N/A	[20, 27, 38]
Serum/Plasma	PPMS	HC	4	58/127	*X*^2^ = 3.47 *P* = 0.325	*I*^2^ = 13.5%	SMD = 0.875 (0.546–1.203)	Z = 5.21 P <0.001	N/A	[20, 27, 38, 44]
Plasma	PPMS	NIND and HC	3	43/132	*X*^2^ = 0.08 *P* = 0.962	*I*^2^ = 0.0%	SMD = 0.660 (0.291–1.028)	Z = 3.51 P <0.001	N/A	[20, 27, 38]
Serum/Plasma	PPMS	NIND and HC	4	58/171	*X*^2^ = 1.36 *P* = 0.715	*I*^2^ = 0.0%	SMD = 0.767 (0.448–1.085)	Z = 4.72 P <0.001	N/A	[20, 27, 38, 44]
Serum/Plasma	CIS	RRMS	3	77/470	*X*^2^ = 9.14 *P* = 0.010	*I*^2^ = 78.1%	SMD = 0.197 (-0.378–0.772)	Z = 0.67 *P* = 0.501	N/A	[27, 38, 44]
Serum/Plasma	CIS	PPMS	3	77/48	*X*^2^ = 1.70 *P* = 0.427	*I*^2^ = 0.0%	SMD = -0.486 (-0.858 - -0.114)	Z = 2.56 *P* = 0.010	N/A	[27, 38, 44]
Serum/Plasma	RRMS	PPMS	5	533/63	*X*^2^ = 21.95 *P <0*.*001*	*I*^2^ = 81.8%	SMD = -0.436 (-1.131–0.259)	Z = 1.23 *P* = 0.219	T = -0.47 *P* = 0.672	[20, 25, 27, 38, 44]
Serum/Plasma	RRMS	SPMS	5	500/108	*X*^2^ = 43.92 *P <0*.*001*	*I*^2^ = 90.9%	SMD = -0.495 (-1.400–0.409)	Z = 1.07 *P* = 0.283	T = -0.46 *P* = 0.674	[20, 25, 27, 38, 43]
Serum/Plasma	RRMS	Progressive MS	6	544/171	*X*^2^ = 45.83 *P <0*.*001*	*I*^2^ = 89.1%	SMD = -0.546 (-1.211–0.118)	Z = 1.61 *P* = 0.107	T = -0.92 *P* = 0.408	[20, 25, 27, 38, 43, 44]
Serum/Plasma	PPMS	SPMS	4	48/102	*X*^2^ = 2.69 *P* = 0.443	*I*^2^ = 0.0%	SMD = 0.217 (-0.133–0.567)	Z = 1.22 *P* = 0.224	N/A	[20, 25, 27, 38]

*patients who were drug naïve or samples were collected after the washout period.

†samples were collected during the remission period or after at least one month of stable disease in progressive types of MS.

**Fig 4:**
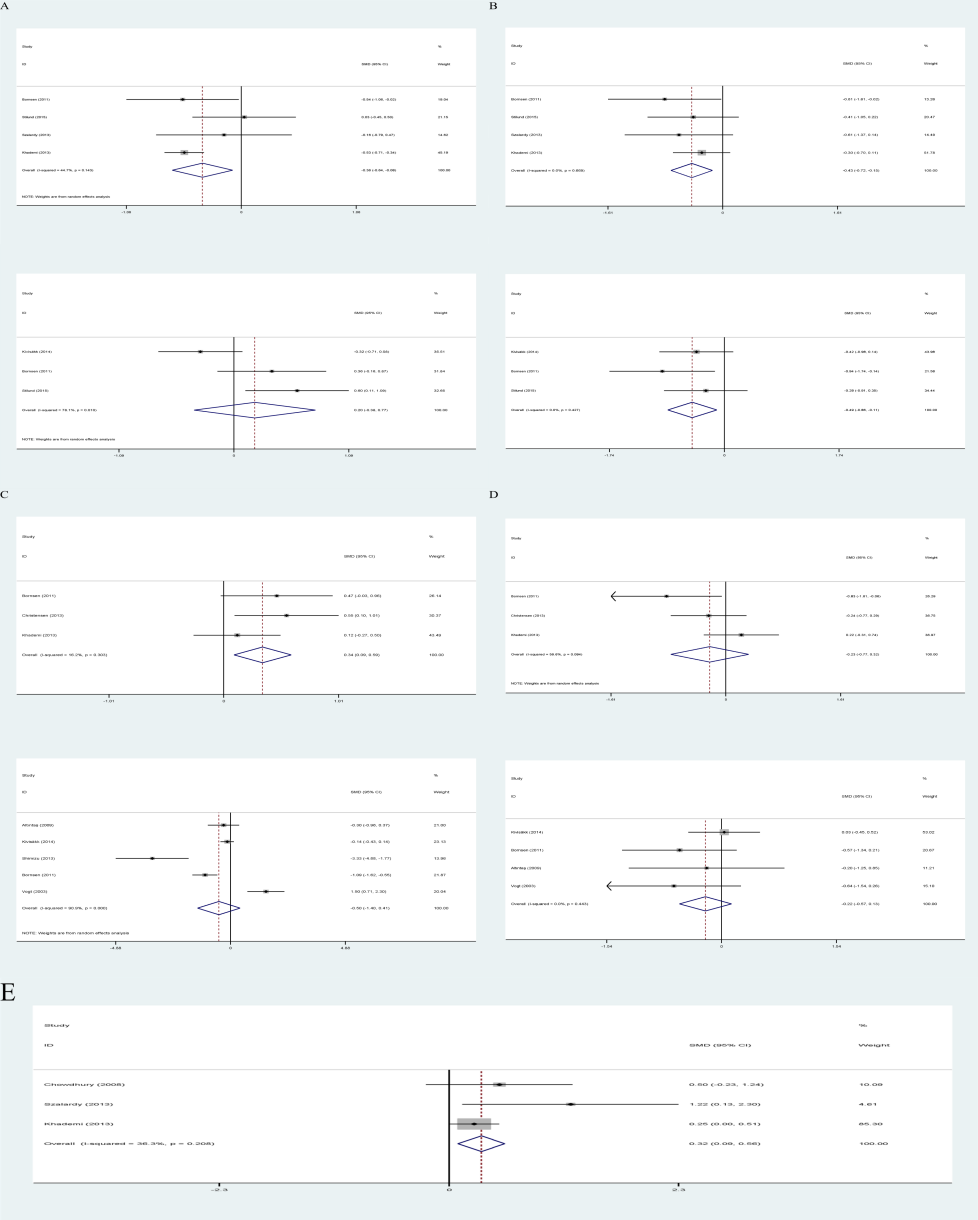
CSF and peripheral blood concentration of OPN; comparisons among subtypes of MS. (A) CIS vs RRMS (first CSF, second peripheral blood). (B) CIS vs PPMS (first CSF, second peripheral blood). (C)RRMS vs SPMS (first CSF, second peripheral blood). (D) SPMS vs PPMS (first CSF, second peripheral blood). (E) Active MS vs Stable MS (only CSF).

In the second set of meta-analyses, we compared the peripheral blood and CSF concentration of OPN between subtypes of MS patients. CIS patients had significantly lower levels of OPN both in their peripheral blood and CSF in comparison to the PPMS patients (Fig 4). CSF concentration of OPN was significantly higher among RRMS patients compared to the CIS patients and SPMS patients (P<0.05, Fig 4). Finally, patients with active MS had significantly higher OPN levels in their CSF compared to patients with stable disease (P = 0.007, Fig 4). The OPN levels did not differ significantly among the other subtypes of MS patients either in CSF or peripheral blood (p>0.05).

As is demonstrated in Tables 2 and 3, heterogeneity was found in majority of meta-analyses; therefore, meta-analysis with a random effects model was applied to the study. Publication bias was evaluated by Egger’s test and funnel plot (S2 and S3 Files), which pointed to no significant bias for most of the meta-analyses except the bold ones in Tables 2 and 3.

## Discussion

Here we present a systematic review and meta-analysis of OPN concentration in MS patients’ peripheral blood and CSF samples for the first time. The results of this study indicate that both peripheral blood and CSF levels of this biomarker are increased among MS patients. Although CSF samples are more sensitive for detecting biomarker levels in MS, blood biomarkers are preferred because they are collected more easily by a less invasive procedure. Therefore, significantly higher levels of OPN in peripheral blood of MS patients compared to controls could be an interesting finding of the present study which emphasizes the clinical applicability of this biomarker even more.

Finding higher levels of OPN in samples from MS patients is in agreement with the T cell mediated nature of the disease [54]. OPN is highly expressed in activated T cells and can modulate the activation pathway by cytokine regulation [16]. Ex vivo studies on T cells from both experimental autoimmune encephalomyelitis (EAE) models and MS patients indicate an increase in the number of OPN receptors on these cells [55]. It was shown that in the presence of human OPN, purified CD4+ T cells from MS patients exert more inflammatory responses compared with healthy controls [55]. Moreover, Braitch et al. reported that OPN levels were positively correlated with the relative presence of Th1 cytokine, IL12p40, in the CSF of patients with MS [28].Chabas *et al*. suggested that OPN may have an important role in MS pathology by Th1 response regulation; they showed that while production of IL-10 was increased in OPN^-/-^ mice, INF-γ and IL-12 productions were diminished compared to OPN^+/+^ ones [21]. Cell survival maintenance is another feature of OPN that may contribute to its role in autoimmune diseases such as MS [16, 56, 57]. It was demonstrated that injecting recombinant OPN to OPN-/- mice reversed the remission phase of EAE and led to disease progression which was proposed to be consequent to enhanced survival of autoreactive T cells mediated by OPN [19]. New evidence suggests that OPN can increase IL-17 production and thereby lead to Th17 differentiation, which is another T cell activation pathway that may induce autoimmunity in MS [55, 58–60].

While OPN levels are significantly higher in CSF of MS patients compared to non-inflammatory controls, we did not detect any significant difference between MS patients and patients with other inflammatory neurological disorders regarding their CSF concentration of OPN. Therefore, we can conclude that presence of any inflammatory process within CNS may lead to increased level of OPN in CSF.

In the present study, we also compared the CSF and peripheral blood levels of OPN among the subtypes of MS patients. The result demonstrates lower CSF and peripheral blood levels of OPN in CIS patients compared to patients with progressive subtypes of MS. This finding supports the possibility for coexistence of neurodegeneration and neuroinflammation in progressive MS [1]. Higher CSF and blood levels of OPN have also been found in common neurodegenerative diseases such as Alzheimer’s disease and Parkinson’s disease; however, high OPN levels cannot be the sole culprit because evidence regarding the neuroprotective effects of OPN also exist [61–63]. We have also shown that CSF concentration of OPN is greater among RRMS patients compared to CIS and SPMS patients. Furthermore, concentrations of OPN in CSF of patients with active MS are significantly increased compared to patients with stable disease. These findings suggest that higher levels of OPN are associated with more active inflammation and highlight the potential of OPN as a prognostic biomarker for patients diagnosed with MS.

In the present meta-analysis, we found a high rate of between-study heterogeneity. The difference in design and sample processing methods of the included studies might be the main cause of this heterogeneity [11]. Sample collection methodology is especially important in peripheral blood biomarker assays because either serum or plasma with different procedures can be used. Although plasma samples are obtained more easily, serum samples are preferred for biomarker detection because of their higher sensitivity [64]. As was mentioned above, differences in study designs are another potential cause of inconsistency among the studies. However, we should not forget that MS is naturally a heterogeneous disease with different subtypes [65]. Even patients from the same category of the disease are not essentially similar to each other due to incongruity of their disease course [65]. Thus, part of this heterogeneity is inevitable and could be a reflection of diversity among patients in their disease activity status; therefore, this might manifest OPN strength in defining the true nature of the disease. The dissimilarity between studies in their control groups, treatment status, and disease activity status are among important sources of heterogeneity, which we tried to attenuate by subgroup analyses [11].

The present study is the first systematic review and meta-analysis of studies which measured peripheral blood and CSF levels of OPN in MS patients and controls. However, we should mention some limitations. Although the existing data strongly suggest that higher levels of OPN are present in peripheral blood and CSF of MS patients compared to the controls, very limited studies were included in most of the subgroup analyses; so to achieve more reliable results, we need more studies to be included in these subgroups. Considerable heterogeneity among the included studies, which we discussed earlier, is another limitation of the present study. Finally, publication bias is a challenging issue in biomarker studies which may affect results of meta-analyses and their reliability [66]. We found publication bias in few meta-analyses of peripheral blood and CSF studies.

In conclusion, the result of this study confirms that increased levels of OPN exist in CSF and peripheral blood of MS patients and strengthens the evidence regarding the clinical utility of OPN as a promising and validated biomarker for MS. In our opinion, OPN can be applicated as diagnostic or predictive, and prognostic biomarker in the clinical setting. An elevated level of OPN in a patient at risk of MS may be suggestive of active inflammation. Given the fact that OPN levels are higher during relapses, we think that by monitoring this biomarker we might be able to predict the disease course. Finally, we propose that developing drugs modulating OPN concentration may be a new treatment strategy for MS.

## Supporting information

S1 File PRISMA 2009 checklist. (DOC)

S2 File Funnel plots for CSF studies. (DOCX)

S3 File Funnel plots for peripheral blood studies. (DOCX)

S1 Appendix Search strategy. (DOCX)

S1 Table Main characteristics of the included studies. (DOCX)
