# Supplementary material for: Osteopontin (OPN) as a CSF and blood biomarker for multiple sclerosis: A systematic review and meta-analysis
Source: PLoS One. 2018 Jan 18;13(1):e0190252. doi: 10.1371/journal.pone.0190252 (PMC5773083; doi:10.1371/journal.pone.0190252)
Supplement: S2 File — (DOCX) [file pone.0190252.s002.docx]

File S2. Funnel plots for CSF studies.

(A) MS patients versus all controls meta-analysis

(B) MS patients versus and NIND patients meta-analysis.

(C) RRMS patients versus HCs and NIND patients meta-analysis.

(D) PPMS patients versus HCs and NIND patients meta-analysis.

(E) RRMS versus PPMS meta-analysis.

(F) RRMS versus Progressive MS meta-analysis.
