# Supplementary material for: Osteopontin (OPN) as a CSF and blood biomarker for multiple sclerosis: A systematic review and meta-analysis
Source: PLoS One. 2018 Jan 18;13(1):e0190252. doi: 10.1371/journal.pone.0190252 (PMC5773083; doi:10.1371/journal.pone.0190252)
Supplement: S3 File — (DOCX) [file pone.0190252.s003.docx]

File S3. Funnel plots for peripheral blood studies.

(A) MS versus HCs meta-analysis.

(B) MS patients versus HCs and NIND patients meta-analysis.

(C) RRMS patients versus HCs meta-analysis.

(D) RRMS patients versus HCs and NIND patients.

(E) RRMS versus PPMS meta-analysis.

(F) RRMS versus SPMS meta-analysis.
