# Supplementary material for: Osteopontin (OPN) as a CSF and blood biomarker for multiple sclerosis: A systematic review and meta-analysis
Source: PLoS One. 2018 Jan 18;13(1):e0190252. doi: 10.1371/journal.pone.0190252 (PMC5773083; doi:10.1371/journal.pone.0190252)
Supplement: S1 Appendix — (DOCX) [file pone.0190252.s004.docx]

**Appendix S1. Search strategy**

**PubMed and Web of Science**

((Osteopontin OR OPN OR Bone sialoprotein I OR BSP-1 OR BSP 1 OR BSP1 OR BSPI OR BNSP OR Early T-lymphocyte activation OR ETA-1 OR ETA 1 OR ETA1 OR ETAI OR Secreted phosphoprotein 1 OR SPP-1 OR SPP 1 OR SPP1 OR SPPI OR Rickettsia resistance OR Ric)) AND (Multiple sclerosis[Title/Abstract] OR MS[Title/Abstract] OR Disseminated sclerosis[Title/Abstract] OR Encephalomyelitis disseminata[Title/Abstract])

**Scopus**

TITLE-ABS-KEY ( "Multiple sclerosis" OR "MS" OR "Disseminated sclerosis" OR "Encephalomyelitis disseminate") AND TITLE-ABS-KEY ( "Osteopontin" OR "OPN" OR "Bone sialoprotein I" OR "BSP1" OR "BSP 1" OR "BSP1" OR "BSPI" OR "BNSP" OR "Early Tlymphocyte activation" OR "ETA1" OR "ETA 1" OR "ETA1" OR "ETAI" OR "Secreted phosphoprotein 1" OR "SPP1" OR "SPP 1" OR "SPP1" OR "SPPI" OR "Rickettsia resistance" OR "Ric" )
