# Supplementary material for: Osteopontin (OPN) as a CSF and blood biomarker for multiple sclerosis: A systematic review and meta-analysis
Source: PLoS One. 2018 Jan 18;13(1):e0190252. doi: 10.1371/journal.pone.0190252 (PMC5773083; doi:10.1371/journal.pone.0190252)
Supplement: S1 Table — (DOCX) [file pone.0190252.s005.docx]

Table S1. Main characteristics of the included studies.

| First author (YOP) | Country | Stuy Population | | Control Group | | Specimen | OPN level (mean±SD) | | Method | Ref |
| --- | --- | --- | --- | --- | --- | --- | --- | --- | --- | --- |
|  |  | MS type (n, male%) | Age (years) | Type (n, male%) | Age (years) |  | MS | Control |  |  |
| Ferret-Sena 2016 | Portugal | RRMS (12, 0%) | 43±12 | HC (9. 0%) | N/A | Plasma | 104.1­±40.6 | 51.1±18 | ELISA | [1] |
| [Strehlow](https://www.ncbi.nlm.nih.gov/pubmed/?term=Strehlow%20F%5BAuthor%5D&cauthor=true&cauthor_uid=27294357) 2016 | Germany | MS (8,38%) | Median (range): 45 (31–60) | HC (13, 62%)  IND (6, 50%)  NIND (9, 56%) | Median (range):  53 (23–81)  58 (42–69)  58 (45–75) | CSF | 181.25±99 | 302.5±83.68  374±80.92  73.75±52.32 | ELISA | [2] |
| Stilund 2015 | Denmark | RRMS (44, 16%)  PPMS (15, 53%)  CIS (27, 26%) | Mean (range): 37 (23–62)  53 (35–64)  37 (16–71) | HC (39, 10%) | Mean (range): 41 (25-57) | Serum | 19.54±8.65  30.72±18.6  26.23±14.43 | 18.05±7.40 | ELISA | [3] |
| Kariya 2015 | Japan | MS (19, 5%) | 34.4 ± 10.8 | OND (14, 14%)  NMO (19, 0%) | 47.7 ± 19.1  47.4 ± 14.7 | CSF | 152.82±27.25 | 139.04±35.45  1554.89±641.75 | ELISA | [4] |
| Ma 2014 | China | RRMS (10, N/A) | N/A | HC (10, N/A) | N/A | Serum | 8.11±3.99 | 3.68±0.67 | ELISA | [5] |
| Kivisäkk 2014 | USA | MS (492, 25%)  RRMS (388, 22%)  SPMS (54, 31.5%)  PPMS (24, 54%)  CIS (26, 15%) | 45.9‌±11.0  44.3±10.3  54±10.5  57.8±10  42.3±9.6 | HC (54, 28%) | 42.4±12.8 | Plasma | 42.4756±17.2425  41.26±13.03  43.22±17.88  42.64±13.28  37.13±12.77 | 36.55±7.66 | ELISA | [6] |
| Iaffalano 2014 | Italy | RRMS  (*Treated*: 49, 24%)  RRMS  (*Not-treated*: 24, 29%) | 34.23±10.12  35.8±10.83 | HC (22, 45%) | 39.18±10.12 | Plasma | 65.42±22.2  67.7±24.23 | 53.2±12.68 | ELISA | [7] |
| Khademi 2013 | Sweden | CIS (169, 26%)  RRMS (389, 29%)  SPMS (54, 39%)  PPMS (28, 50%) | 35.9(16–65)  34.3 (17–73)  54.6 (35–81)  51.7 (35–67) | IND (223, 26%)  NIND (203, 28%) | 49.6 (13–83)  41.1 (19–82) | CSF | 116.9±36.37  140.4±47.6  134.9±31.58  127.6±35.41 | 144.6±54.14  111.8±40.14 | ELISA | [8] |
| Edwards 2013 | UK and Germany | MS (40, 26%) | Median (range): 42.5 (18-70) | NIND (22, 5%)  IND (8, 62.5%) | Median (range):  N/A  67 (38-72) | CSF | (365.33±49.20) * 10^-3^ | (235.08±57.18)* 10^-3^  (338.60±48.60)* 10^-3^ | ELISA | [9] |
| Szalardy 2013 | Hungary | **MS^*^** (74, 38%)  CIS (23, N/A)  RRMS (17, N/A)  PPMS (10, N/A) | Median (IQR):  35.2 (18.3)  N/A  N/A  N/A | **NIND^†^** (30, 50%) | Median (IQR):  36.3 (19.4) | CSF | 140.6±139.56  123.9±106.96  147.1±184.07  202.3±168 | 77.9±50.96 | ELISA | [10] |
| Shimizo 2013 | Japan | MS (17, 35%)  RRMS (11, N/A)  SPMS (6, N/A) | 38.3 ±10.4 | HC (20, 30%)  NMO (16, 6%) | 26.3 ± 2.8  43.3 ± 10.3 | Plasma | MS:  *Relapse* (69.24±36.85)  *Remission* (61.64±16.31)  RRMS (53.39±4.54)  SPMS (93.28±19.76( | HC:  (33.76±26.56)  NMO:  *Relapse* (76.86± 30.18)  *Stable* (52.72±23.16) | ELISA | [11] |
| Romme Christensen 2013 | Sweden and Denmark | RRMS (36, 44%)  SPMS (40, 47.5%)  PPMS (21, 48%) | Median (IQR):  34 (31–40)  51 (44–57)  48 (38–53) | NIND (20, 55%) | Median (IQR):  53 (39–65) | CSF | 237.14±130  171.43±107.14  192.86±30 | 114.29±54.29 | ELISA | [12] |
| Wen 2012 | China | RRMS (51, 29%) | 36.49± 5.15 | NIND (48, 43.75%) | 35.20± 11.71 | Serum  CSF | 43.13±25.71  199.75±92.78 | 34.29±16.95  41.17±14.21 | ELISA | [13] |
| Assadi 2011 | Iran | RRMS (35, 23%) | 31.57± 7.26 | HC (38, 21%) | 30± 6.12 | Serum | 41.2±2.35 | 37.67±2.46 | ELISA | [14] |
| Bornsen 2011 | Sweden and Denmark | **CIS^ǂ^** (25, 32%)  **RRMS^§^** (41, 44%)  PPMS (9, 44%)  **SPMS^¶^** (28, 46%) | N/A | HC (24, 37.5%)  NIND (44, 41%) | N/A  N/A | Plasma  CSF | 47.9±15.69  43±12.15  64.35±21.73  56±11.71  150.33±99.26  216±132.59  221.67±38.52  163.33±77.04 | 44.76±8.2  125.67±66.67 | ELISA | [15] |
| Vogt 2010 | Netherlands | RRMS (36, N/A) | N/A | HC (20, N/A) | N/A | Plasma | 380.95±114.75 | 333.95±51.74 | ELISA | [16] |
| Khademi 2009 | Sweden and Denmark | **RRMS^ǁ^** (22, 36%) | Mean (range):  40.6 (22–56 ) | NIND (25, 28%) | Mean (range):  37.6 (25–60) | CSF | 261.11±86.42 | 126.86±55.56 | ELISA | [17] |
| Altıntaş 2009 | Turkey | MS (50, 34%)  RRMS (33, N/A)  SPMS (12, N/A)  PPMS (5, N/A) | 37.4±10.8 | HC (30, 20%) | 36.5± 8.8 | Plasma | 15.9±36.2  11.3±32.1  21.7±42.7  30.4±45.6 | 155.4±81.8 | ELISA | [18] |
| Chowdhury 2008 | USA | MS (30, 40%) |  | OND (36, 39%) |  | CSF | 7400±6400 | 5800±4400 | ELISA | [19] |
| Braitch 2008 | UK | MS (27, 37%) | 42.1± 10.1 | IND (11, 64%)  NIND (24, 25%) | 50.5± 15.8  42.1±18.7 | CSF  Plasma | 415±186  380±236 | 563±411  286±150  386±271  377±121 | ELISA | [20] |
| Chiocchetti 2005 | Italy | MS (71, N/A) | N/A | HC (81, N/A) | N/A | Serum | 211.77±104.9 | 138.23±73.53 | ELISA | [21] |
| Vogt 2003 | Netherlands | RRMS (30, 17%)  PPMS (10, 40%)  SPMS (10, 50%) | 39 ± 6.2  51.2 ± 12.6  47.4 ± 6.9 | HC (10, N/A) | N/A | Plasma | 285.7±60  235.7±64.5  198.6±50.6 | 195.7±52.2 | ELISA | [22] |

ELISA= enzyme-linked immunosorbent assay. CIS= clinically isolated syndrome. CSF= cerebrospinal fluid. MS= multiple sclerosis. N/A= not available. OPN= osteopontin. PPMS= primary progressive multiple sclerosis. RRMS= relapsing-remitting multiple sclerosis. SPMS= secondary progressive multiple sclerosis. YOP= year of publication. *= OPN levels were measured in 50 samples. †= OPN levels were measured in 19 samples. ǂ= OPN levels were measured in 24 samples. §= OPN levels were measured in 38 samples. ¶= OPN levels were measured in 26 samples. ǁ= OPN levels were measured in 20 samples.

1. Ferret-Sena, V., E.S.A. Maia, and A. Sena, *Natalizumab Treatment Modulates Peroxisome Proliferator-Activated Receptors Expression in Women with Multiple Sclerosis.* 2016. **2016**: p. 5716415.

2. Strehlow, F., et al., *Osteopontin in cerebrospinal fluid as diagnostic biomarker for central nervous system lymphoma.* 2016. **129**(1): p. 165-71.

3. Stilund, M., et al., *Biomarkers of inflammation and axonal degeneration/damage in patients with newly diagnosed multiple sclerosis: contributions of the soluble CD163 CSF/serum ratio to a biomarker panel.* PLoS One, 2015. **10**(4): p. e0119681.

4. Kariya, Y., et al., *Increased cerebrospinal fluid osteopontin levels and its involvement in macrophage infiltration in neuromyelitis optica.* BBA Clin, 2015. **3**: p. 126-34.

5. Ma, N., et al., *BAFF maintains T-cell survival by inducing OPN expression in B cells.* Mol Immunol, 2014. **57**(2): p. 129-37.

6. Kivisakk, P., et al., *Evaluation of circulating osteopontin levels in an unselected cohort of patients with multiple sclerosis: relevance for biomarker development.* Mult Scler, 2014. **20**(4): p. 438-44.

7. Iaffaldano, P., et al., *The improvement of cognitive functions is associated with a decrease of plasma Osteopontin levels in Natalizumab treated relapsing multiple sclerosis.* Brain Behav Immun, 2014. **35**: p. 176-81.

8. Khademi, M., et al., *Intense inflammation and nerve damage in early multiple sclerosis subsides at older age: a reflection by cerebrospinal fluid biomarkers.* PLoS One, 2013. **8**(5): p. e63172.

9. Edwards, L.J., et al., *Increased levels of interleukins 2 and 17 in the cerebrospinal fluid of patients with idiopathic intracranial hypertension.* Am J Clin Exp Immunol, 2013. **2**(3): p. 234-44.

10. Szalardy, L., et al., *Evaluating biomarkers of neuronal degeneration and neuroinflammation in CSF of patients with multiple sclerosis-osteopontin as a potential marker of clinical severity.* J Neurol Sci, 2013. **331**(1-2): p. 38-42.

11. Shimizu, Y., et al., *Plasma osteopontin levels are associated with disease activity in the patients with multiple sclerosis and neuromyelitis optica.* J Neuroimmunol, 2013. **263**(1-2): p. 148-51.

12. Romme Christensen, J., et al., *CSF inflammation and axonal damage are increased and correlate in progressive multiple sclerosis.* Mult Scler, 2013. **19**(7): p. 877-84.

13. Wen, S.R., et al., *Increased levels of IL-23 and osteopontin in serum and cerebrospinal fluid of multiple sclerosis patients.* J Neuroimmunol, 2012. **244**(1-2): p. 94-6.

14. Assadi, M., et al., *Correlation of circulating omentin-1 with bone mineral density in multiple sclerosis: the crosstalk between bone and adipose tissue.* PLoS One, 2011. **6**(9): p. e24240.

15. Bornsen, L., et al., *Osteopontin concentrations are increased in cerebrospinal fluid during attacks of multiple sclerosis.* Mult Scler, 2011. **17**(1): p. 32-42.

16. Vogt, M.H., et al., *Increased osteopontin plasma levels in multiple sclerosis patients correlate with bone-specific markers.* Mult Scler, 2010. **16**(4): p. 443-9.

17. Khademi, M., et al., *The effects of natalizumab on inflammatory mediators in multiple sclerosis: prospects for treatment-sensitive biomarkers.* Eur J Neurol, 2009. **16**(4): p. 528-36.

18. Altintas, A., et al., *The role of osteopontin: a shared pathway in the pathogenesis of multiple sclerosis and osteoporosis?* J Neurol Sci, 2009. **276**(1-2): p. 41-4.

19. Chowdhury, S.A., J. Lin, and S.A. Sadiq, *Specificity and correlation with disease activity of cerebrospinal fluid osteopontin levels in patients with multiple sclerosis.* Arch Neurol, 2008. **65**(2): p. 232-5.

20. Braitch, M., et al., *Increased osteopontin levels in the cerebrospinal fluid of patients with multiple sclerosis.* Arch Neurol, 2008. **65**(5): p. 633-5.

21. Chiocchetti, A., et al., *Osteopontin gene haplotypes correlate with multiple sclerosis development and progression.* J Neuroimmunol, 2005. **163**(1-2): p. 172-8.

22. Vogt, M.H., et al., *Elevated osteopontin levels in active relapsing-remitting multiple sclerosis.* Ann Neurol, 2003. **53**(6): p. 819-22.
